# Serum neurofilament light at diagnosis: a prognostic indicator for accelerated disease progression in Parkinson’s Disease

**DOI:** 10.1038/s41531-024-00768-1

**Published:** 2024-08-21

**Authors:** Camilla Christina Pedersen, Anastasia Ushakova, Guido Alves, Ole-Bjørn Tysnes, Kaj Blennow, Henrik Zetterberg, Jodi Maple-Grødem, Johannes Lange

**Affiliations:** 1 The Centre for Movement Disorders, Stavanger University Hospital, Stavanger, Norway; 2 Department of Chemistry, Bioscience and Environmental Engineering, University of Stavanger, Stavanger, Norway; 3 Section of Biostatistics, Department of Research, Stavanger University Hospital, Stavanger, Norway; 4 Department of Neurology, Stavanger University Hospital, Stavanger, Norway; 5 Department of Clinical Medicine, University of Bergen, Bergen, Norway; 6 Department of Neurology, Haukeland University Hospital, Bergen, Norway; 7 Department of Psychiatry and Neurochemistry, Institute of Neuroscience and Physiology, The Sahlgrenska Academy at the University of Gothenburg, Mölndal, Sweden; 8 Clinical Neurochemistry Laboratory, Sahlgrenska University Hospital, Mölndal, Sweden; 9 Paris Brain Institute, ICM, Pitié-Salpêtrière Hospital, Sorbonne University, Paris, France; 10 Neurodegenerative Disorder Research Center, Division of Life Sciences and Medicine, and Department of Neurology, Institute on Aging and Brain Disorders, University of Science and Technology of China and First Affiliated Hospital of USTC, Hefei, P.R. China; 11 Department of Neurodegenerative Disease, UCL Institute of Neurology, London, UK; 12 UK Dementia Research Institute at UCL, London, UK; 13 Hong Kong Center for Neurodegenerative Diseases, Hong Kong, China; 14 Wisconsin Alzheimer’s Disease Research Center, University of Wisconsin School of Medicine and Public Health, University of Wisconsin-Madison, Madison, Wisconsin USA

**Keywords:** Parkinson's disease, Prognostic markers, Parkinson's disease

## Abstract

Neurofilament light chain (NFL) is elevated in neurodegenerative diseases, including Parkinson’s disease (PD). This study aimed to investigate serum NFL in newly diagnosed PD and its association with cognitive and motor decline over 10 years. Serum NFL levels were measured in PD patients and controls from the ParkWest study at diagnosis (baseline) and after 3 and 5 years. Mixed-effects regression analyzed changes in NFL and the association with annual changes in MMSE and UPDRS-III scores over 10 years. PD patients had elevated serum NFL at all visits and a faster annual increase over 5 years compared to controls (0.09 pg/mL per year; *p* = 0.029). Higher baseline NFL predicted faster cognitive decline β −0.77 transformed MMSE; *p* = 0.010), and a 40% NFL increase predicted future motor decline (β 0.28 UPDRS-III; *p* = 0.004). Elevated serum NFL in early PD is linked to faster cognitive and motor impairment, suggesting its prognostic value in PD biomarker panels.

## Introduction

Neurofilament light chain (NFL) is a subunit of the neurofilament protein and is a well-established biomarker of neuroaxonal degeneration in a range of neurological disorders, including Parkinson’s disease (PD)^1,2^. With the development of ultrasensitive detection methods, NFL can be assessed in easily accessible sample types, such as blood (i.e., plasma or serum)^3^.

While blood NFL has been associated with worsening cognitive^4–6^ and motor^7,8^ function in some studies, others have found contrasting results^9–11^. Additionally, there is a scarcity of longitudinal measurements of NFL in larger prospective cohorts, particularly in early, population-based PD, which limits our understanding of how the change in NFL correlates with disease progression. Gathering further insights into the dynamic evolution of NFL in PD, especially in its early stages, is crucial for understanding its potential prognostic value in PD.

The aim of this study was to investigate longitudinal measurements of serum NFL in a prospective study of newly diagnosed PD and the potential associations with the development of cognitive and motor decline over the first 10 years of the disease.

## Results

### Demographic and clinical characteristics

In the present study, we included a total of 392 participants, including 190 patients with PD and 202 controls. Demographic and clinical characteristics are displayed for patients with PD (*N* = 172, 90.5%) and controls (*N* = 194, 96.0%) who had measurements of serum NFL at their baseline visit (Table 1). Patients had fewer years of education (*p* = 0.002) and lower MMSE scores than controls (*p* = 0.001) at the baseline visit. The remaining participants (18 patients with PD and 8 controls) provided serum samples only at the year 3 and/or 5 visits (See Supplementary Figs. 1 and 2 for further details).

**Table 1:** Demographic and clinical characteristics of controls and patients included in the study

Clinical variables^a^	PD	Controls	*P*-value
*N* Total	172	194	
Male, *N* (%)	104 (60.5)	104 (53.6)	0.186
BL Age, years, Mean (SD)	68.2 (9.0)	66.5 (9.7)	0.065
Time since diagnosis, days	40.0 (20.5–61.0)	–	–
Time since first PD motor symptoms, years	1.8 (1.1–3.1)	–	–
BL Education, years	11 (8–13)	12 (9–15)	**0.002**
BL UPDRS III	21.0 (15.0–32.0)	–	–
BL HY	2.0 (1.0–2.5)	–	–
BL MMSE score	28 (27–29)	29 (28–30)	**0.001**
BL NFL, pg/mL	17.8 (12.9–25.1)	15.2 (10.6–21.9)	**0.003**
Year 3 NFL, pg/mL	*N* = 146, 20.4 (14.5–31.3)	*N* = 144, 16.3 (11.1–24.4)	<**0.001**
Year 5 NFL, pg/mL	*N* = 137, 20.9 (16.3–33.1)	*N* = 150, 17.2 (12.7–25.5)	<**0.001**
Percent change in NFL BL to Year 5, %	*N* = 124, +36.1 (16.6–62.8)	*N* = 142, +20.1 (4.7–37.8)	<**0.001**

Bold values are statistically significant at *p* < 0.05.

*N* number of participants, *BL* baseline, *SD* standard deviation, *PD* Parkinson’s disease, *UPDRS III* Unified Parkinson Disease Rating Scale Part III, *HY* Hoehn and Yahr stage, *MMSE* mini-mental state examination, *NFL* neurofilament light chain.

^a^Median (first to third quartiles) are reported unless otherwise stated.

At baseline, there was a significant association of serum NFL with age in both patients with PD (β 7.80; 95% CI 6.71–8.90; *p* < 0.001) and controls (β 8.65; 95% CI 7.30–10.00; *p* < 0.001). No associations were found between baseline NFL and sex, UDPRS III, or MMSE in either group (all *p* > 0.05).

### NFL comparisons between diagnostic groups

Serum NFL was measured at baseline (PD = 172, controls = 194), year 3 (PD = 146, controls = 144) and year 5 (PD = 137, controls = 150). Patients had higher levels of NFL than controls at each visit (Table 1; Supplementary Fig. 3). After adjustment for age and sex, these differences were borderline not significant at baseline (β 0.11 for log2-transformed NFL; 95% CI 0.00–0.23; *p* = 0.052), with patients estimated to have 1.3 pg/mL (1.2–1.4) higher NFL than controls. By year 3, the regression estimate increased to 0.25 for log2 NFL (95% CI 0.12–0.38; *p* < 0.001), and the estimated difference in NFL was 3.2 pg/mL (95% CI 3.0–3.4). By year 5, the regression estimate rose further to 0.33 for log2 NFL (95% CI 0.21–0.46; *p* < 0.001), with an estimated difference of 4.6 pg/mL (95% CI 4.3–5.0) higher NFL in patients compared to controls. In sensitivity analysis, using an age-matched subgroup of patients and controls (see “Methods”), patients continued to exhibit increased NFL levels at each visit compared to controls. Specifically, at baseline, the difference was estimated to be 2.5 pg/mL (95% CI 0.9–4.2), *p* = 0.003; at 3 years, the difference was 2.6 pg/mL (95% CI 0.6–4.7), *p* = 0.012; and at 5 years, the difference was 5.1 pg/mL (95% CI 3.0–7.4), *p* < 0.001.

**Fig. 1:**
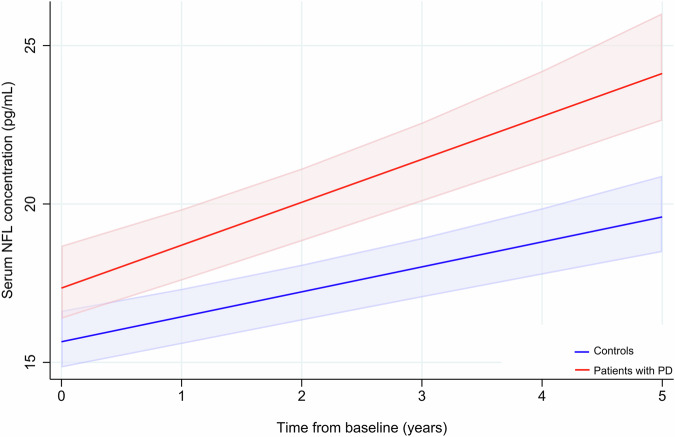
Prediction of NFL levels over 5 years. Predicted NFL levels with 95% confidence intervals from baseline to 5 years for controls (blue) and patients with PD (red). NFL neurofilament light chain.

At the group level, patients with PD had a larger increase in percent change in NFL from baseline to year 5 than controls (Table 1). This difference remained significant after adjustment for age and sex (β 14.66; 95% CI 7.46–21.87; *p* < 0.001). The change in NFL over time was next investigated using linear mixed-effects models (LME). Over the first 5 years of the study, NFL increased in both patients with PD and controls: The annual change of log2 transformed NFL for controls was 0.07 (95% CI 0.05–0.08, *p* < 0.001) and patients with PD had higher levels of log2 transformed NFL at baseline by 0.16 (95% CI 0.04–0.28, *p* = 0.009) and experienced a faster increase in serum NFL of 0.03 units (95% CI 0.00–0.05, *p* = 0.029) per year. This corresponds to an adjusted mean difference of 1.8 pg/mL at baseline, followed by an increase of 0.50 pg/mL per year, compared to controls (Fig. 1).

### Serum NFL and cognitive decline

We assessed the annual rate of global cognitive impairment measured using MMSE over 10 years in both controls and patients with PD. While there was no significant annual change in controls (β −0.24 transformed MMSE points; 95% CI −0.55 to 0.06; *p* = 0.121), MMSE scores decreased in patients with PD (β −1.87; 95% CI −2.28 to −1.46; *p* < 0.001) with a predicted drop from 84.4 to 62.2 transformed points over 10 years (Supplementary Fig. 4A). This corresponds to an estimated decrease in MMSE score from 29 to 26 points.

**Fig. 2:**
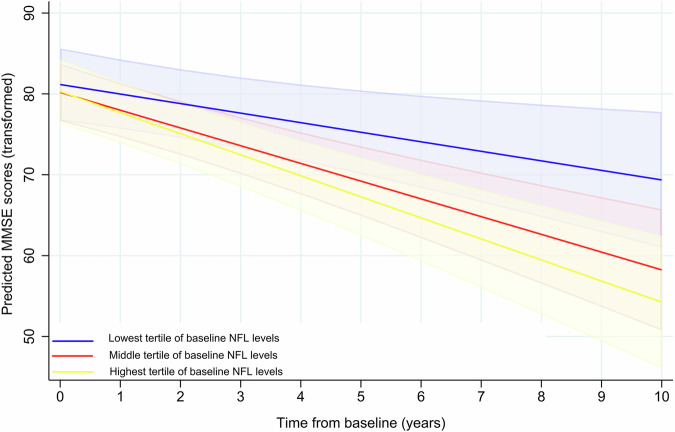
Prediction of MMSE over 10 years. Baseline serum NFL prediction of transformed MMSE scores, with 95% confidence intervals. Patients were categorized into lowest (blue), middle (red), and highest (yellow) NFL tertiles. MMSE mini-mental State Examination, NFL neurofilament light chain.

We then examined whether baseline serum NFL can predict changes in MMSE scores in patients, measured over 10 years. For every one-unit increase in log_2_-transformed NFL, patients had a predicted annual decrease of 0.77 transformed MMSE points (*p* = 0.010; Table 2). To further explore the association between baseline serum NFL and MMSE, patients were categorized into tertiles based on lowest (<13.1 pg/mL), middle (13.1–20.5 pg/mL), and highest (>20.5 pg/mL) baseline serum NFL (Fig. 2). Compared to patients in the lowest NFL group, patients in the highest NFL tertile were predicted to experience a faster annual decline in MMSE score (*p* = 0.022; Table 2). The average predicted drop over 10 years for patients in the lowest tertile corresponds to an estimated change in MMSE score from 29 to 27 points. Patients in the highest tertile experience an average predicted change in MMSE score from 29 to 24 points. There were no significant differences between the predictions for the lowest and middle tertile.

**Table 2:** Association of baseline NFL with clinical assessment scores over 10 years, in patients with PD categorized into tertiles of NFL

Clinical assessments	Main effect^a^ β (95% CI)	*P*	Interaction with time^a^ β (95% CI)	*P*
MMSE^**b**^				
Overall NFL^c^	−0.05 (−3.17 to 3.08)	0.977	−0.77 (−1.36 to −0.19)	**0.010**
Tertiles of NFL				
Lowest NFL	Ref		Ref	
Middle NFL	−0.98 (−6.69 to 4.74)	0.738	−1.01 (−2.19 to 0.16)	0.091
Highest NFL	−0.88 (−7.50 to 5.75)	0.795	−1.42 (−2.64 to 0.21)	**0.022**
UPDRS				
Overall NFL^c^	2.25 (0.00 to 4.50)	0.050	0.21 (−0.19 to 0.61)	0.296

Bold values are statistically significant *p* < 0.05. Models are adjusted for age at baseline and sex. Education was included as a covariate for the MMSE model. NFL cut-offs for tertile categorization: Lowest < 13.1 pg/mL; Middle 13.1–20.5 pg/mL; Highest > 20.5 pg/mL.

*MMSE* mini-mental state examination, *NFL* neurofilament light chain, *UPDRS III* Unified Parkinson Disease Rating Scale Part III, *CI* confidence interval, *Ref* reference.

^a^Main effect indicates the effect of NFL levels on the intercept and the interaction with time indicates the effect of NFL levels on slope (annual change per year) of the model.

^b^MMSE and NFL were transformed as described in the “Methods” section.

^c^Overall NFL indicates that NFL was included in the model as a continuous variable.

We further assessed whether the rate of change in NFL was associated with annual changes in MMSE. The percent change in NFL was calculated for patients with NFL measurements at baseline and year 5 (*n* = 124; 72.1%). One patient was excluded from the analysis because of missing MMSE scores at and after year 5. While a 40% increase in NFL from baseline to year 5 was associated with a 2.60-point decrease in transformed MMSE (95% CI −3.79 to −1.41; *p* < 0.001) at year 5, a 40% increase in NFL had no effect on the predicted change in MMSE from year 5 through year 10 (β 0.30; 95% CI −0.73 to 0.14; *p* = 0.179).

While LMMs provide valuable insights into the overall trends and average rates of cognitive decline in the cohort, cognitive decline in PD is complex, is not necessarily constant, and can exhibit variable patterns over time. Patients were divided into three groups based on their performance in the MMSE across multiple visits (as described in “Methods”). Baseline NFL level between the groups was different (*p* = 0.001), and post hoc comparison revealed that the group that remained “stable cognitively normal” over follow-up (*n* = 68, 44.7%) had lower baseline NFL compared to either those with a “variable cognitive course” (*n* = 25, 16.4%) or those with a pattern of “stable cognitive decline” (*n* = 59, 38.8%), *p* = 0.007 or *p* = 0.001, respectively.

### Serum NFL and motor decline

We assessed the annual rate of motor impairment in patients with PD. Patients experienced a mean annual increase of 1.34 UPDRS III points (95% CI 1.04–1.64; *p* < 0.001), with a predicted increase from 20.5 to 33.9 points over 10 years (Supplementary Fig. 4B). No association was found between baseline NFL and the annual change in UPDRS III (Table 2). To account for the effect of medication, we repeated the analysis with adjustment for repeated measures of total levodopa equivalent dose (LED) at each year and found that the association between baseline NFL and UDPRS III remained not significant (data not shown).

We also evaluated whether the percent change in NFL could predict changes in UPDRS III. At year 5, a 40% increase in NFL from baseline to year 5 was associated with a 1.69-point higher UPDRS III score (95% CI 1.01–2.36; *p* < 0.001). A 40% increase in NFL was also associated with a mean annual increase of an additional 0.28 points in UPDRS III (95% CI 0.10–0.47) from year 5 through year 10. This corresponds to a predicted increase in UPDRS III from 26.7 to 36.9 points for a male patient of average age who experiences a 40% increase in NFL from baseline to year 5. When adjusting for LED at each year, the association remained significant (data not shown).

## Discussion

We found elevated baseline serum NFL levels and greater changes in NFL over time in a large number of newly diagnosed patients with PD compared to controls from the ParkWest longitudinal cohort. Additionally, for patients, we also found associations between serum NFL levels at diagnosis and changes in NFL over the first 5 years of disease with subsequent cognitive and motor decline. These findings were based on up to 10 years of prospective clinical data.

In our study, levels of serum NFL were higher in patients with PD compared to controls at each visit. Both groups experienced an increase in NFL over 5 years, with a faster increase in patients. This is in line with previous findings for serum NFL^12–14^. In addition, studies investigating NFL in plasma and CSF have also found the same trend^14–16^. CSF is considered the gold standard biofluid for biomarkers in neurological disorders due to its proximity to the central nervous system and thus, its ability to reflect changes occurring in the brain. However, serum NFL levels have been found to be well correlated with those in CSF^17^. This highlights serum NFL as an accessible biomarker for PD diagnosis and neurodegenerative brain changes related to the development and progression of the disease. Further, our findings of elevated levels at PD diagnosis could indicate that changes in NFL levels may begin in the prodromal phase.

We found that higher baseline NFL predicted faster cognitive decline. Our population-based results support the results obtained in a PPMI study using up to 8-year follow-up data, with a mean follow-up of 6.37 ± 1.84 years, from de novo patients with PD^6^. While an association between baseline serum NFL and measures of cognitive decline has also been found in two other longitudinal cohorts, they were no longer significant after adjustment for age, sex, and other covariates^10,18^. However, in these studies, patients had disease durations of 11.0^10^ or 1.3 years^18^, respectively, and had shorter follow-up, which makes a direct comparison with our study of newly diagnosed patients challenging. A suggested minimum clinically important difference (MCID) for MMSE ranges from 1.6 to 2 points^19^. Even though the predicted effect of baseline NFL on the mean annual change in MMSE in our study is small, the predicted change in MMSE over 10 years is clinically meaningful. Additionally, we observed meaningful differences between the projected changes in MMSE in patients with the highest levels of baseline NFL compared to those with the lowest levels of baseline NFL. Therefore, our findings indicate the potential use of serum NFL as a prognostic biomarker for cognitive decline in patients with PD.

The association between serum NFL and motor decline was also investigated in our study. Baseline NFL showed no association with motor decline, similar to findings of others^7,10,20^. However, a greater increase in NFL over the first 5 years of the disease predicted subsequent annual increases in UPDRS III scores. Similarly, the PPMI cohort calculated change rates in serum NFL from baseline to year 3 and found that the rate of change in NFL was associated with a predicted annual increase in UPDRS III scores^7^.

There are limitations to our study. For the longitudinal measurements of NFL, not every subject provided a serum sample at all visits, limiting the number of available cases. However, the sample size at year 5 was still large with 137 patients and 150 controls. Patients with PD are prone to falls, putting them at risk of head injuries, which can cause intermediate increases in NFL levels. We were unable to assess the history of falls close to sampling dates to account for this. Also, patients were on symptomatic treatment and assessed in the on-state at follow-up visits. To control for this, we adjusted for dopaminergic treatment in the models used for the UPDRS III analyses.

Our study has considerable strengths, including a large sample set with repeated measures of NFL, and the use of mixed models to analyze the longitudinal effects of NFL on clinical features. We also used both cross-sectional and follow-up data to explore the role of NFL at specific time points and its influence on clinical features of PD over 10 years and used the MMSE as an evaluation tool for cognitive impairment. While the MoCA has been shown to be more sensitive to identify early cognitive impairment, evidence suggests that the MMSE might be better suited to detect longitudinal cognitive changes in PD^21^. Standard pre-analytical procedures were used to obtain and store serum samples, and an ultrasensitive method was used to detect NFL^21^. All sample analyses were also conducted in a well-recognized lab. Finally, a notable strength of our study is that the ParkWest cohort is a well-defined prospective and population-based cohort of patients with incident PD, who were diagnosed according to standardized criteria. This is particularly relevant because, unlike clinic-based studies that may include age-unrepresentative subjects, population-based studies offer a more representative sample of real-world PD populations^22,23^. Such cohorts provide vital information, especially concerning age-dependent outcomes like disease progression.

In conclusion, we show that NFL is associated with cognitive and motor decline in patients with PD. This indicates that NFL has potential as a biomarker for the progression of PD. NFL may be further assessed for its prognostic performance by being included in a panel of other PD-related biomarkers.

## Methods

### Study participants

One-hundred and ninety patients with PD and 202 normal controls were included from the ParkWest study. ParkWest is a Norwegian population-based, longitudinal, multicenter study of newly diagnosed patients with PD^24^. All participants were recruited between 2004 and 2006 from the southwest of Norway. All patients fulfilled the United Kingdom Brain Bank criteria^25^ at the final visit. Five patients were not drug-naïve at least 14 days prior to the baseline visit, and of these, two patients were on a levodopa medication. The control group was recruited from the same population and consisted of spouses and friends of patients, as well as members of public social organizations for the elderly. All participants signed written informed consent. The Regional Committee for Medical and Health Research Ethics in Western Norway approved the study.

### Clinical assessments

Patients were assessed with an extensive, uniform examination program at baseline and during follow-up. Motor severity was assessed using the Unified Parkinson’s Disease Rating Scale part 3 (UPDRS III)^26^, disease stage using the Hoehn and Yahr (H&Y) staging^27^, and global cognition using the mini-mental state examination (MMSE)^28^. Follow-up assessments were included for annual visits for UPDRS III and at years 1, 3, 5, 7, and 9 for MMSE. The mean follow-up was 9.0 ± 0.22 years (range 8.5–10.1 years). An overview of the included visits and number of participants at each can be found in Supplementary Fig. 1.

### Serum sampling, storage, and NFL measurements

Blood samples were collected on the same day as clinical assessments at baseline and at years 3 and 5. Serum samples were prepared as previously described^29^. An overview of available serum samples can be found in Supplementary Fig. 2. Serum NFL concentration was measured by single molecule array (SIMOA) technology on an HD-1 Analyzer (Quanterix, Billerica, MA), as previously described in detail^30^.

### Statistical analysis

STATA 17.0 was used for all analyses. Demographic and clinical data were evaluated for normality using Q-Q plots. Continuous data with normal distribution were reported using the mean and standard deviation (SD), and between-group differences were assessed using the Student’s *t*-test. Continuous data that were not normally distributed were summarized using the median with the 25th and 75th percentiles, and the Mann–Whitney *U*-Test was used to evaluate differences between groups. For categorical variables, between-group differences were assessed using the chi-squared test. NFL levels were not normally distributed and were therefore log_2_ transformed. In sensitivity analysis, age matching was conducted using the R package MatchIt, with all participants grouped into 2-year age groups. For each patient, a matching control was selected from the same age group, resulting in a dataset comprising 165 patients and 165 matching controls.

Using an MMSE score of ≤26 to indicate mild cognitive impairment or cognitive changes that might be suggestive of cognitive decline, patients were categorized into three groups based on the variation of their MMSE scores across multiple visits. Only patients with three or more visits and baseline NFL were included. The groups were: (1) “Stable cognitively normal”, comprising patients scoring 27–30 at every visit; (2) “variable cognitive course”, comprising patients registering a score of ≤26 at any visit but then scoring 27–30 at a subsequent visit, and; (3) “Stable cognitive decline”, including patients registering a score of ≤26 at any visit, and that being either their last visit or they retained a score of ≤26 at all subsequent visits. Between group differences in baseline NFL was measured using Kruskal–Wallis Test with post hoc comparison.

Robust linear regression was used to evaluate the association between PD diagnosis and either log_2_ transformed NFL levels at each time point, or percent changes in NFL, and between baseline log_2_ transformed NFL levels and clinical variables. All analyses were adjusted for age and sex. Additionally, years of education at baseline was included in the MMSE analyses. The predicted values (pg/mL) of NFL based on the regression coefficients for Log2-transformed NFL were estimated using margins command in STATA at the corresponding visit for patients and controls, with all other covariates fixed at their means.

Linear mixed-effects models (LME) were used to study the dynamics of NFL and establish its relation to MMSE and UPDRS III scores. All models were adjusted for age at baseline, sex, and time. Additionally, the models that examined MMSE were adjusted for years of education at baseline. Random effects were patient identifiers (intercepts) and time points (slopes). All models used first-order autoregressive residual covariance structure. For all models investigating cognitive decline, MMSE was normalized as described by Philipps et al. to reduce the ceiling/floor effects and curvilinearity of the raw scores^31^. For further exploration and visualization of the predicted slopes, patients were categorized into tertiles based on baseline NFL levels. LME were also used to analyze the associations between the percent change in NFL from baseline to year 5 and the subsequent annual change in MMSE or UPDRS III. The median percent change in NFL was 36.1% (IQR 16.6–62.8), so the percent change in NFL was rescaled to investigate the effects of a 40% change in NFL. For all models, reported marginal predictions were calculated using the margins command in STATA. Predicted plots were generated using the margins plot command. For the models examining the association between a 40% change in NFL and MMSE or UPDRS III, the margins command yielded scores for a male of average age who experiences a 40% increase in NFL from baseline to year 5.

For all analyses, 2-tailed *p*-values <0.05 are considered significant. Adjustment for multiple testing was not performed.

### Supplementary information

Supplemental Material

## Data Availability

Anonymized data are available upon request to qualified investigators for the purposes of replicating procedures and results.
