# Supplementary material for: Serum neurofilament light at diagnosis: a prognostic indicator for accelerated disease progression in Parkinson’s Disease
Source: NPJ Parkinsons Dis. 2024 Aug 21;10:162. doi: 10.1038/s41531-024-00768-1 (PMC11336184; doi:10.1038/s41531-024-00768-1)
Supplement: Supplementary file 1 — Supplemental Material [file 41531_2024_768_MOESM1_ESM.pdf]

Supplementary Information

	Overall	MMSE	UPDRS III	NFL
Baseline	190 Patients<br>202 Controls	190 Patients<br>202 Controls	190 Patients	172 Patients<br>194 Controls
Year 1	184 Patients<br>191 Controls	183 Patients<br>191 Controls	184 Patients	
Year 3	172 Patients<br>178 Controls	168 Patients<br>178 Controls	172 Patients	146 Patients<br>144 Controls
Year 5	157 Patients<br>162 Controls	151 Patients<br>162 Controls	157 Patients	137 Patients<br>150 Controls
Year 7	143 Patients<br>150 Controls	136 Patients<br>148 Controls	143 Patients	
Year 9	125 Patients<br>139 Controls	118 Patients<br>139 Controls	125 Patients	

Supplementary Figure 1. Flowchart of the number of study participants at each visit.

The overall attendance at each visit, and the number of participants assessed by The Mini Mental State Examination (MMSE) or the Unified Parkinson's Disease Rating Scale part III (UPDRS III), or with serum neurofilament light chain (NFL) measurements are shown.

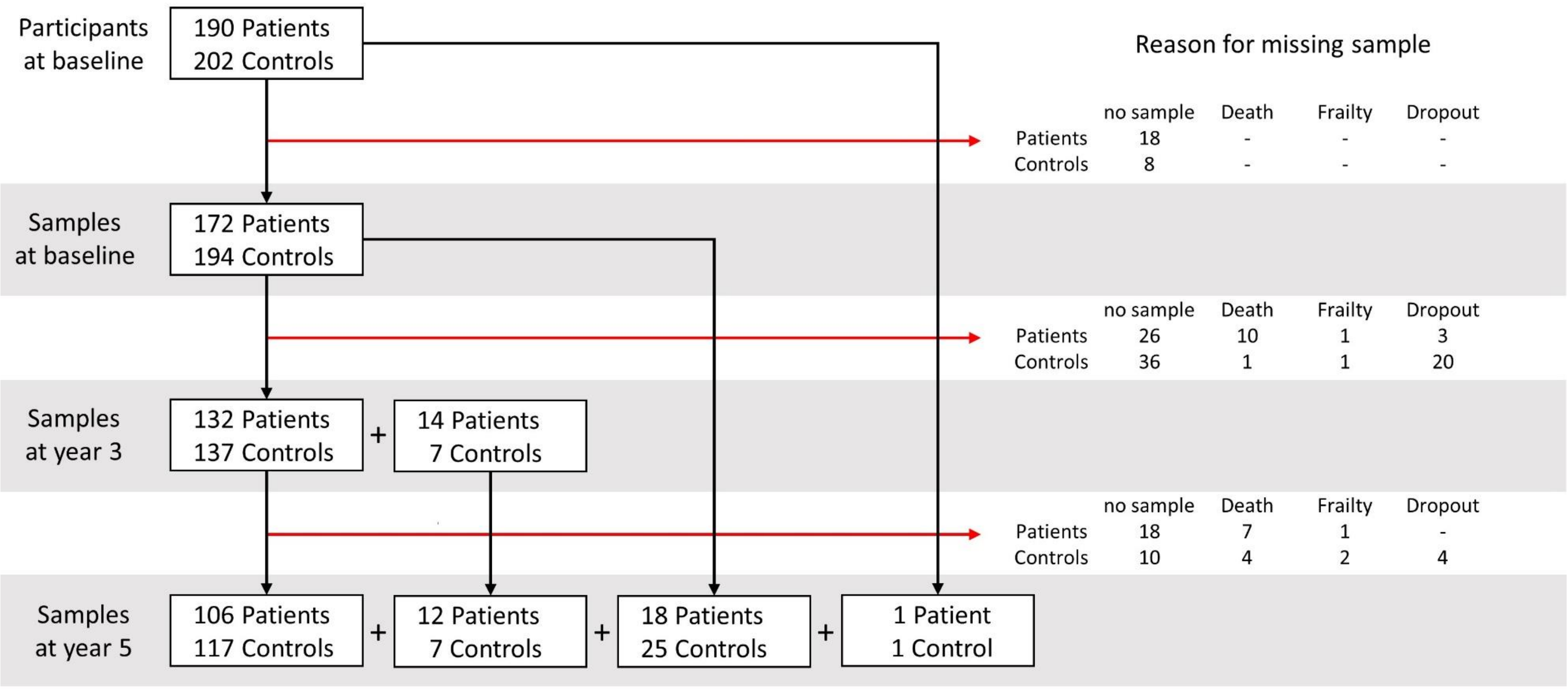

Supplementary Figure 2. Flowchart showing the availability of serum samples.

The number of participants eligible at baseline is shown, along with the number of serum samples available at each visit. The number of samples that are only available from baseline, year 3, or year 5 are indicated. Information regarding the reason for missing samples and the number of missing samples is provided on the right.

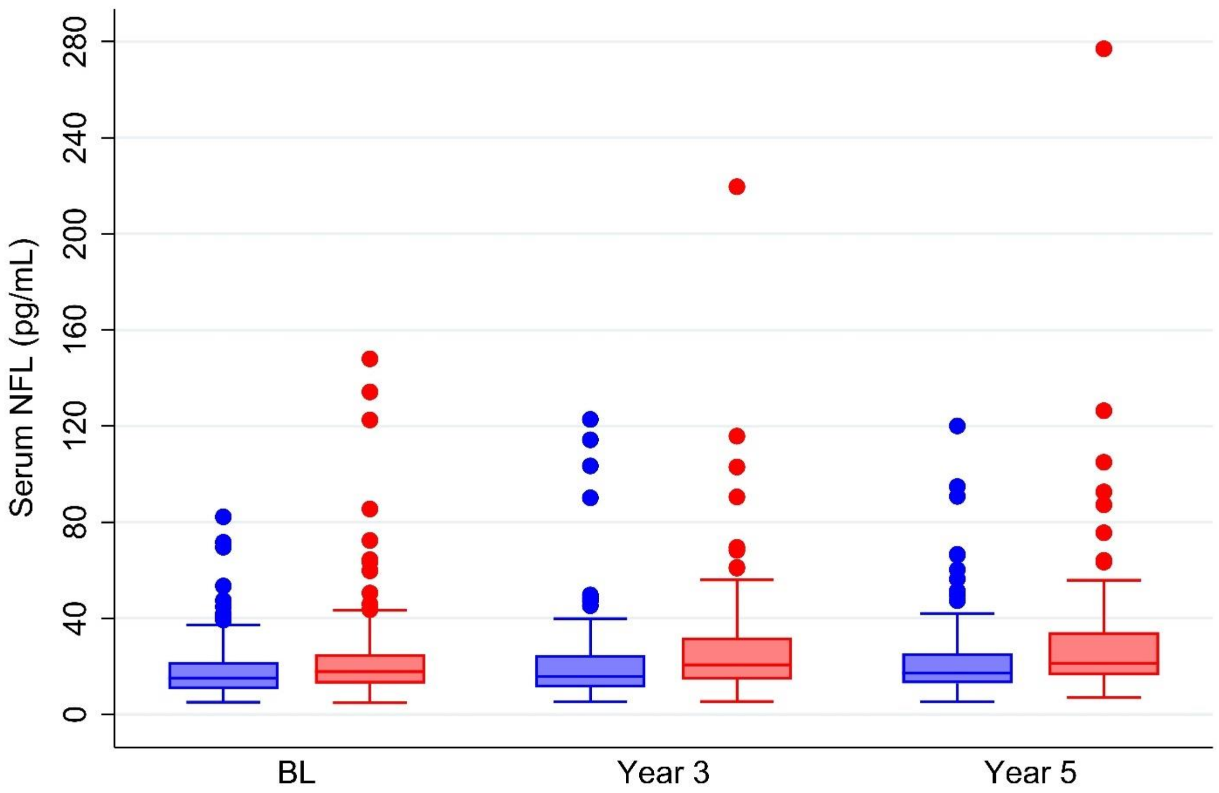

Supplementary Figure 3. Levels of serum NFL at baseline, year 3, and year 5 for controls (blue) and patients with PD (red). Outliers are represented by circles.

Abbreviations; NFL: Neurofilament light chain; BL: Baseline

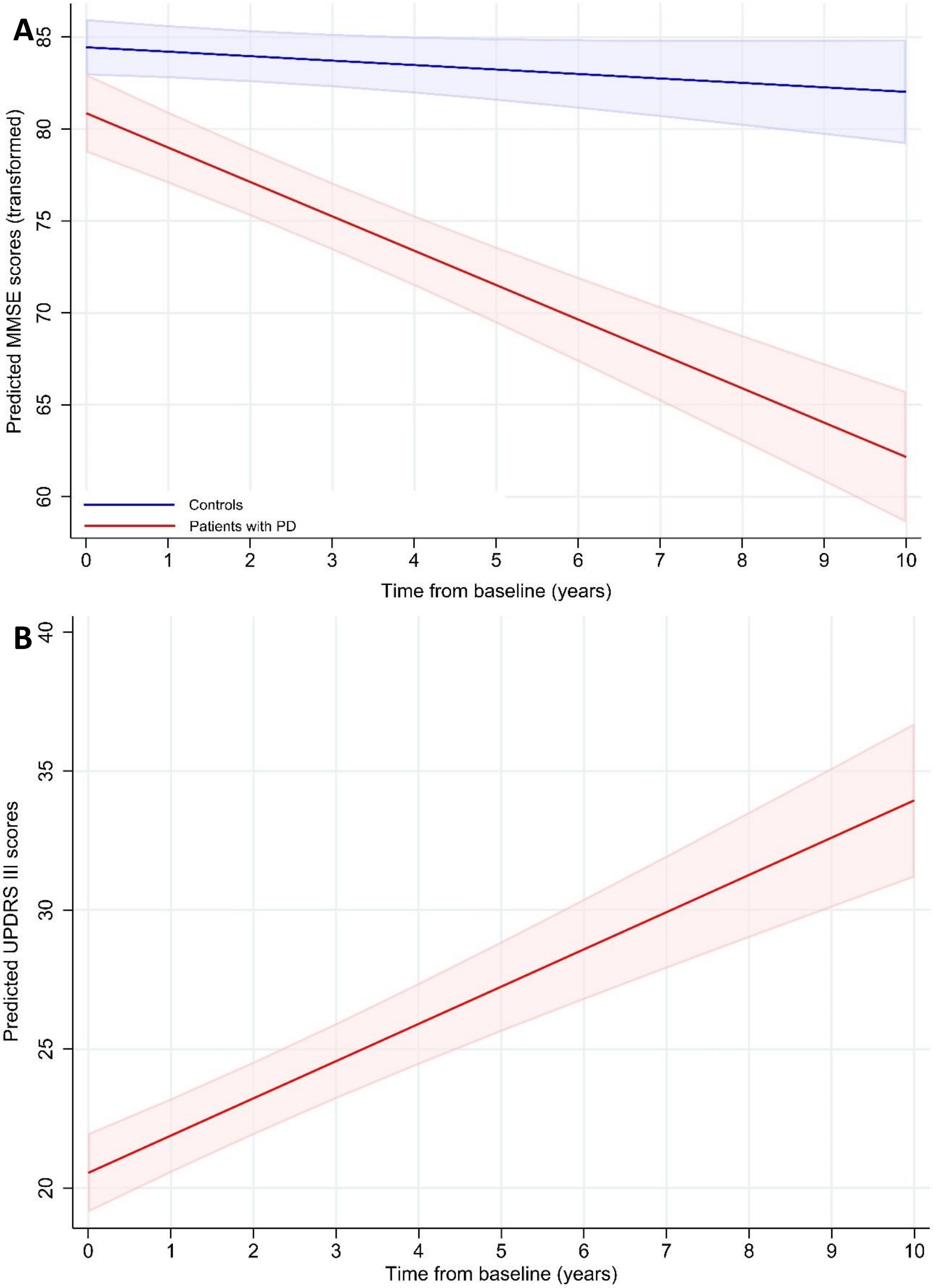

Supplementary Figure 4. Prediction of clinical scores over time. **(A)** Transformed Mini-Mental State Examination (MMSE) scores and **(B)** Unified Parkinson's Disease Rating Scale (UPDRS) III scores in controls (blue) and patients (red). MMSE scores were transformed before analysis, as described in the Methods.
